# Double-stapled intracorporeal anastomosis for minimally invasive right and left colectomies: a standardized technical approach

**DOI:** 10.1007/s00464-026-13360-w

**Published:** 2026-10-01

**Authors:** Richard Fortunato, Selim Gebran, Reem Saab, James T. McCormick

**Affiliations:** 1 Division of Colon and Rectal Surgery, Department of Surgery, Allegheny Health Network, 320 E North Ave, Suite 261, Pittsburgh, PA 15212 USA; 2 Department of Obstetrics and Gynecology, Allegheny Health Network, Pittsburgh, PA USA

**Keywords:** Ileocolonic anastomosis, Double-stapled technique, Intracorporeal anastomosis, Robotic surgery, Colorectal surgery, Surgical technique, Standardization

## Abstract

**Background:**

Minimally invasive surgery (MIS) for colorectal procedures has become increasingly common, with intracorporeal anastomosis (ICA) gaining traction due to its documented patient benefits. However, widespread adoption and teaching of ICA can be challenging, often due to the reliance on advanced intracorporeal suturing skills. This technical note describes a standardized, fully stapled intracorporeal side-to-side anastomosis technique for minimally invasive right and left colectomies, aiming to simplify its execution and improve reproducibility.

**Technique:**

The procedure begins with deliberate port placement and patient positioning tailored for robotic access. Following full bowel mobilization and mesenteric division, anti-mesenteric stay sutures are placed to facilitate optimal alignment. Enterotomies are carefully created, and a double-stapled intracorporeal anastomosis is performed using sequentially oriented 60-mm enteric stapler loads. The technique emphasizes precise tissue handling, proper stapler orientation, and visual confirmation of perfusion.

**Conclusion:**

This standardized, fully stapled approach offers a reproducible and efficient solution to performing intracorporeal side-to-side intestinal anastomosis. By eliminating the need for intracorporeal hand-sewn closure and leveraging familiar stapler-based principles, this technique aims to lower the technical barrier to adoption and facilitate its teaching across both robotic and laparoscopic platforms. Future studies are warranted to investigate the learning curve and clinical outcomes of this standardized technique.

**Supplementary Information:**

The online version contains supplementary material available at https://doi.org/10.1007/s00464-026-13360-w.

Over the past two decades, the preferred approach to colorectal surgery has progressively shifted toward minimally invasive surgery (MIS). This transition has been driven by its association with reduced intraoperative blood loss, lower postoperative complication rates, earlier return of bowel function (ROBF), shorter hospital stays (LOS), and faster return to normal activity compared to open procedures [1–3]. Robotic platforms have further advanced MIS adoption by offering enhanced three-dimensional visualization, tremor filtration, a “3rd arm” assistant port, and superior instrument articulation. These capabilities have enabled the development of novel procedures and refinement of existing laparoscopic MIS techniques, including the intracorporeal anastomosis (ICA) [4–8].

ICA is increasingly favored over extracorporeal anastomosis (ECA) due to numerous reported benefits, including potentially reduced surgical site infections, and lower rates of incisional hernia and ileus [9, 10]. This is particularly relevant in patients with challenging anatomy, prior abdominal surgery, or elevated body mass index (BMI) [5, 7, 8, 11–14]. While various sutureless anastomotic techniques have been previously described [15–18], the technical challenges associated with mastering and teaching intracorporeal anastomosis, particularly the learning curve of intracorporeal suturing, can limit its widespread adoption [17, 19, 20]. The inability to manually inspect the anastomosis and the uncertainty surrounding consistent reproducibility are also significant concerns that must be addressed for surgeons to confidently adopt ICA [21].

To address these concerns and facilitate broader adoption, we have developed a standardized approach to a fully stapled ICA. This technique eliminates the need for intracorporeal hand-sewn suturing by applying the familiar principles of the double-stapled extracorporeal anastomosis (ECA) Barcelona technique to an intracorporeal setting [22]. This technical note details our standardized methods and procedural steps for performing a fully stapled side-to-side intracorporeal anastomosis for both right and left colectomies.

## Surgical technique

The operative methodology for performing our fully stapled intracorporeal side-to-side anastomosis for a minimally invasive right or left colectomy follows a standardized sequence, beginning with precise port placement and patient positioning (Table 1). Robotic port placement for intracorporeal anastomosis in both right and left colectomy utilizes a non-traditional layout, optimizing visualization and stapler trajectory.

**Fig. 1:**
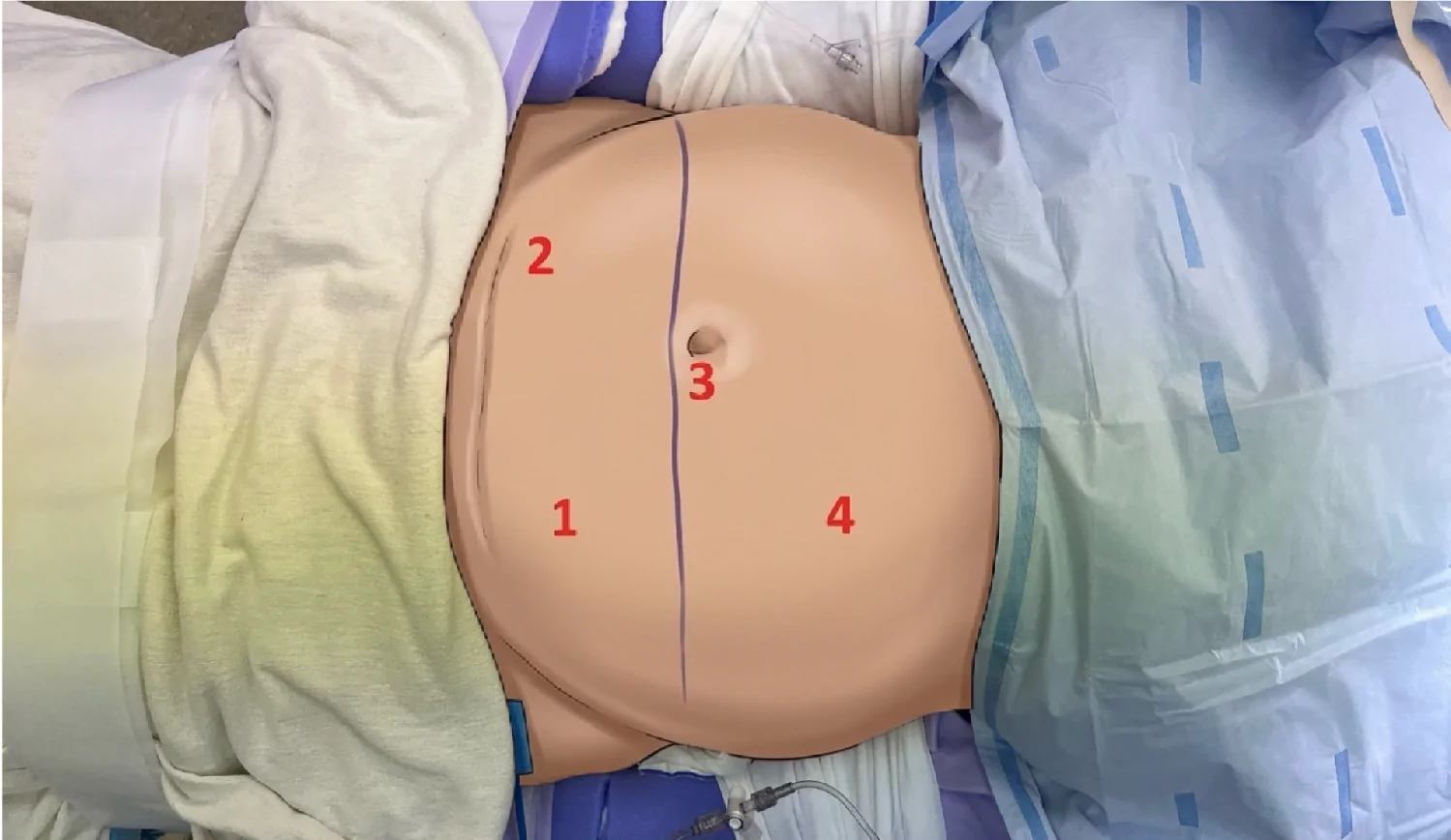
Right colectomy port placement locations with corresponding robotic arms. Port 1 (8-mm left arm), port 2 (12-mm assist arm and stapler), port 3 (8-mm, camera) and port 4 (8 mm, right arm)

**Fig. 2:**
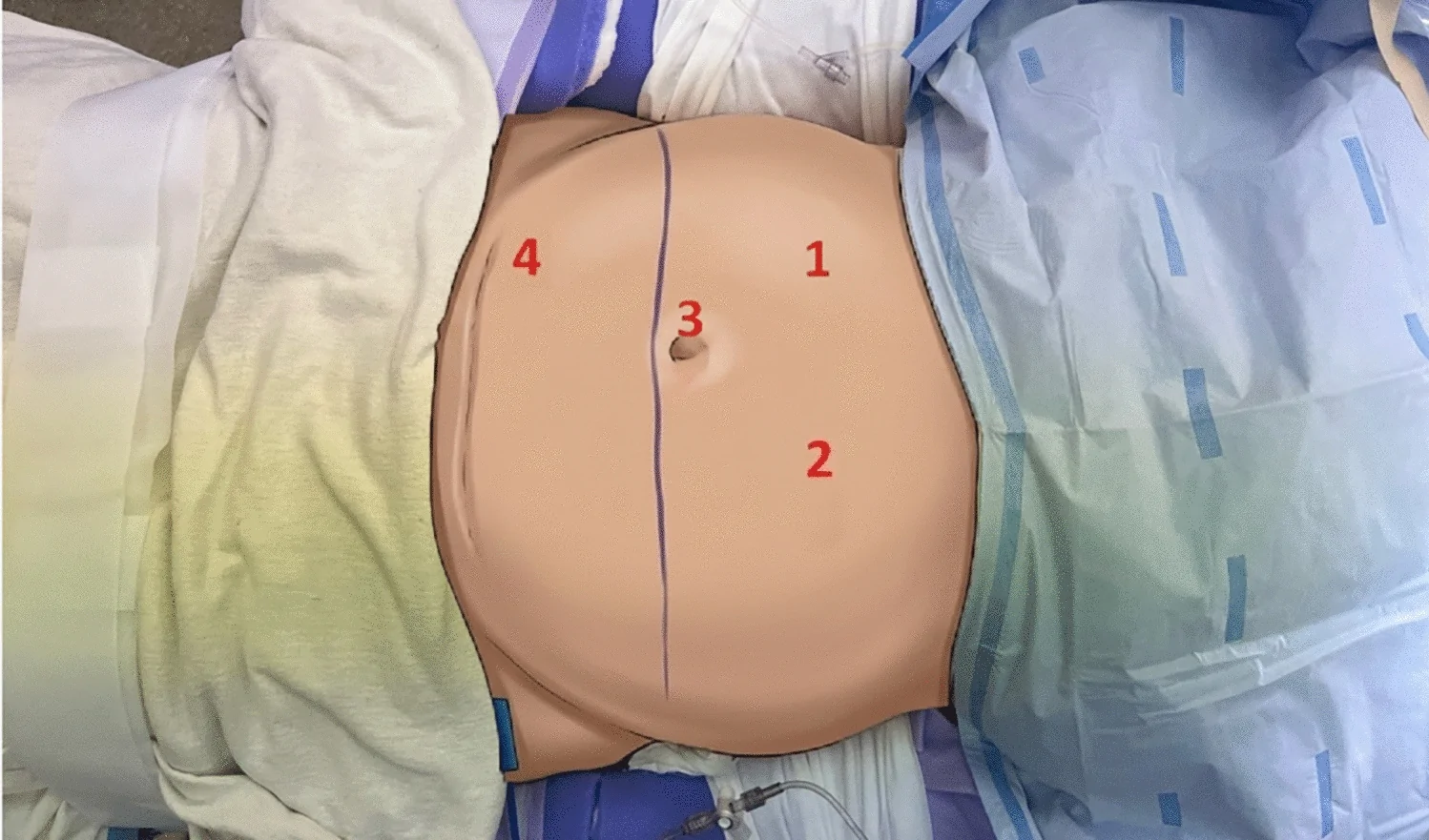
Left colectomy port placement locations with corresponding robotic arms. Port 1 (12-mm left arm and stapler), port 2 (12-mm assist arm and stapler), port 3 (8 mm, camera) and port 4 (8 mm, right arm)

- Right colectomy (enterocolonic anastomosis): The technique begins with a periumbilical 8-mm camera port. Two additional 8-mm robotic arm ports are placed in the left upper and left lower quadrants for the working arms. A 12-mm right-lower-quadrant (RLQ) port is used for the third/assist arm and for stapler access (Fig. 1).
- Left colectomy (colocolonic anastomosis): A similar configuration is used, with an 8-mm camera port at the umbilicus and an 8-mm port in the right lower quadrant. However, 12-mm ports are placed in the left upper quadrant (LUQ) and right upper quadrant (RUQ) to accommodate the initial anastomotic stapler firing and the subsequent perpendicular closure firing, respectively (Fig. 2).

**Table 1:** Port placements and positioning

Colectomy type	Port placement details	Patient position
Right colectomy (enterocolonic anastomosis)	Periumbilical 8-mm camera port Two additional 8-mm robotic arm ports in the left upper and left lower quadrants (working arms) A 12-mm right-lower-quadrant (RLQ) port for third/assist arm and stapler access	Left-side down, slight Trendelenburg position
Left colectomy (colocolonic anastomosis)	Periumbilical 8-mm camera port One 8-mm port in the right lower quadrant (working arm) A 12-mm left upper quadrant (LUQ) port for the first stapler firing A 12-mm right upper quadrant (RUQ) port for the perpendicular closure stapler firing	Right-side down, slight Trendelenburg position

**Fig. 3:**
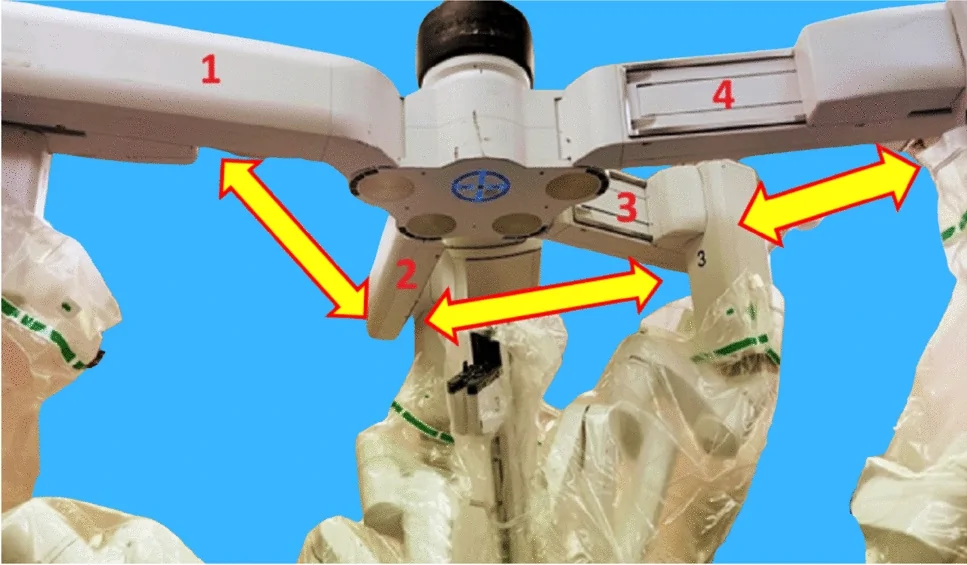
Opening the arms allows for increased range of motion and decreased external collisions

**Fig. 4:**
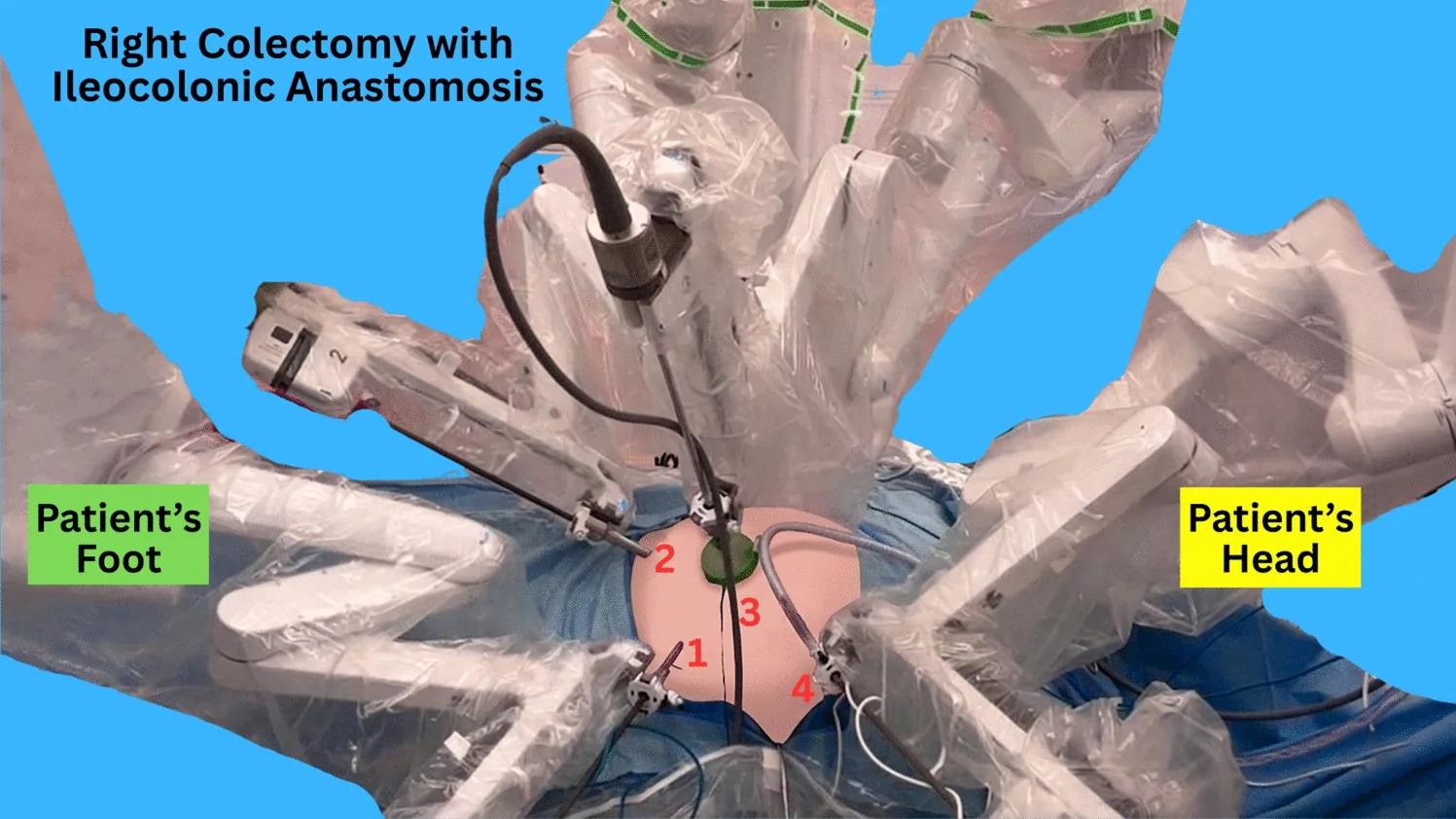
After docking, manually point each arm toward the right abdomen and adjust to avoid collisions

**Fig. 5:**
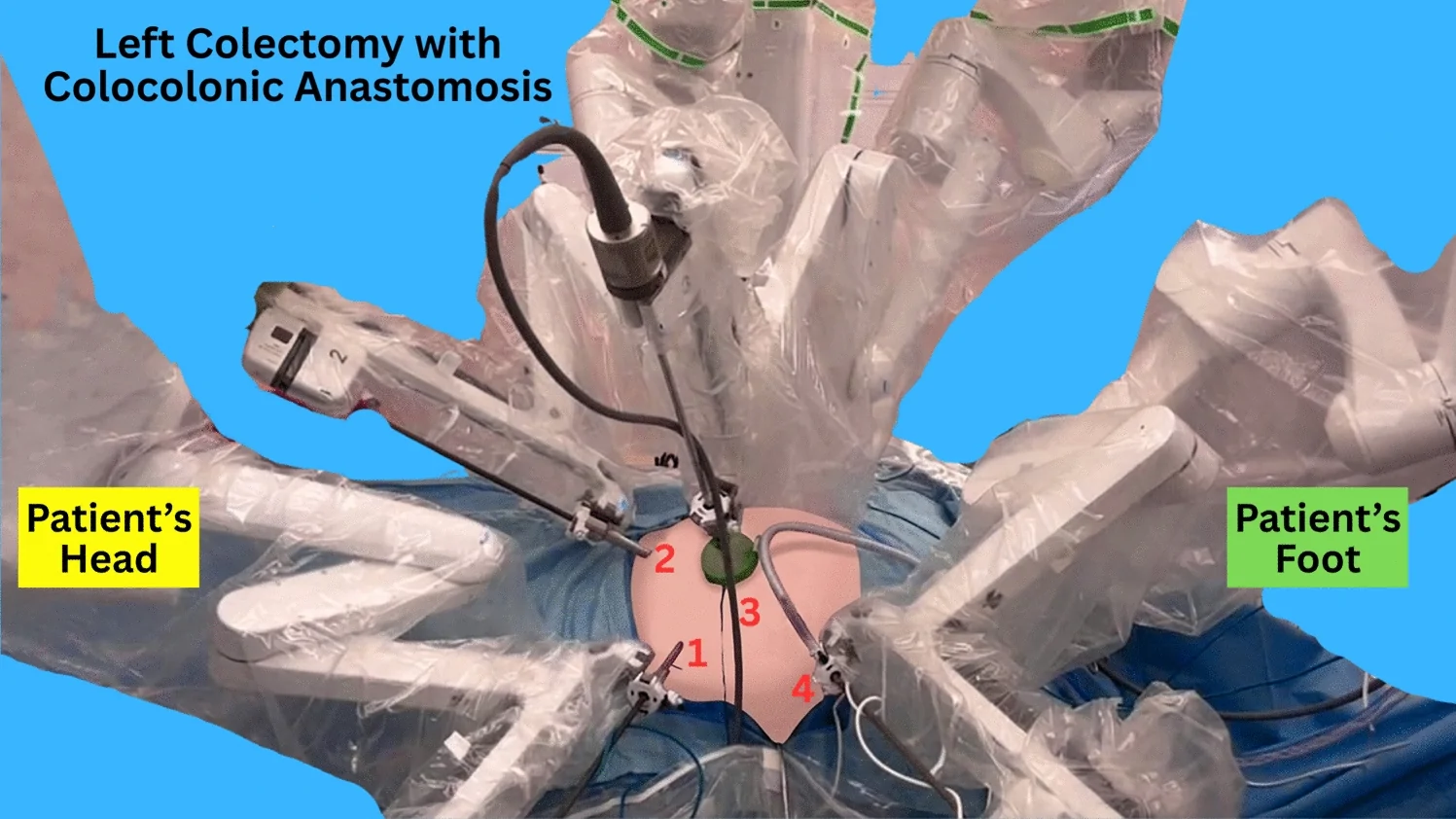
Robot arm positioning in left colectomy is mirror image of the right colectomy with the patient laying in the opposite direction

**Fig. 6:**
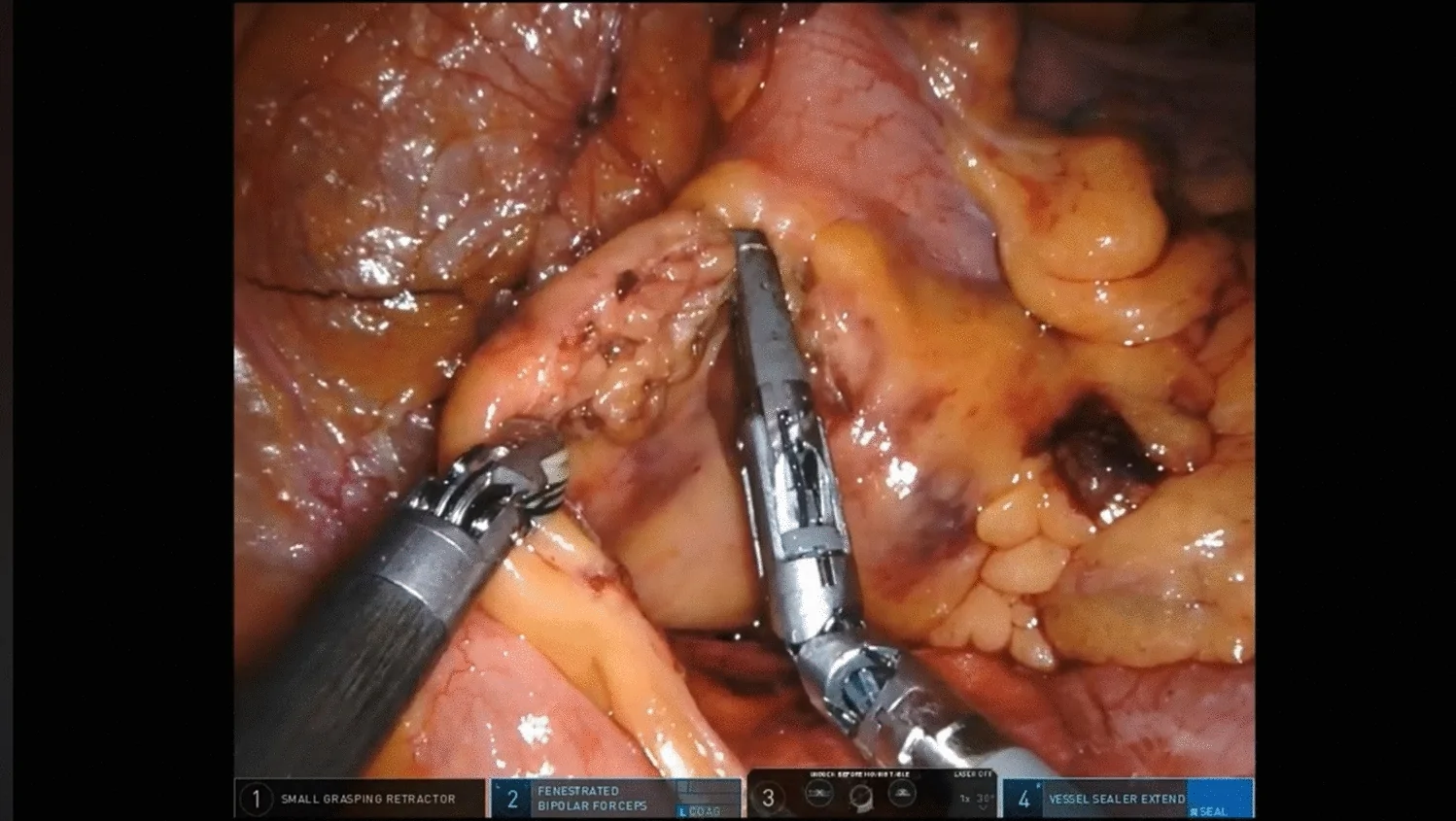
Robotic right colectomy: clearing the ileal mesentery completely to the small bowel

**Fig. 7:**
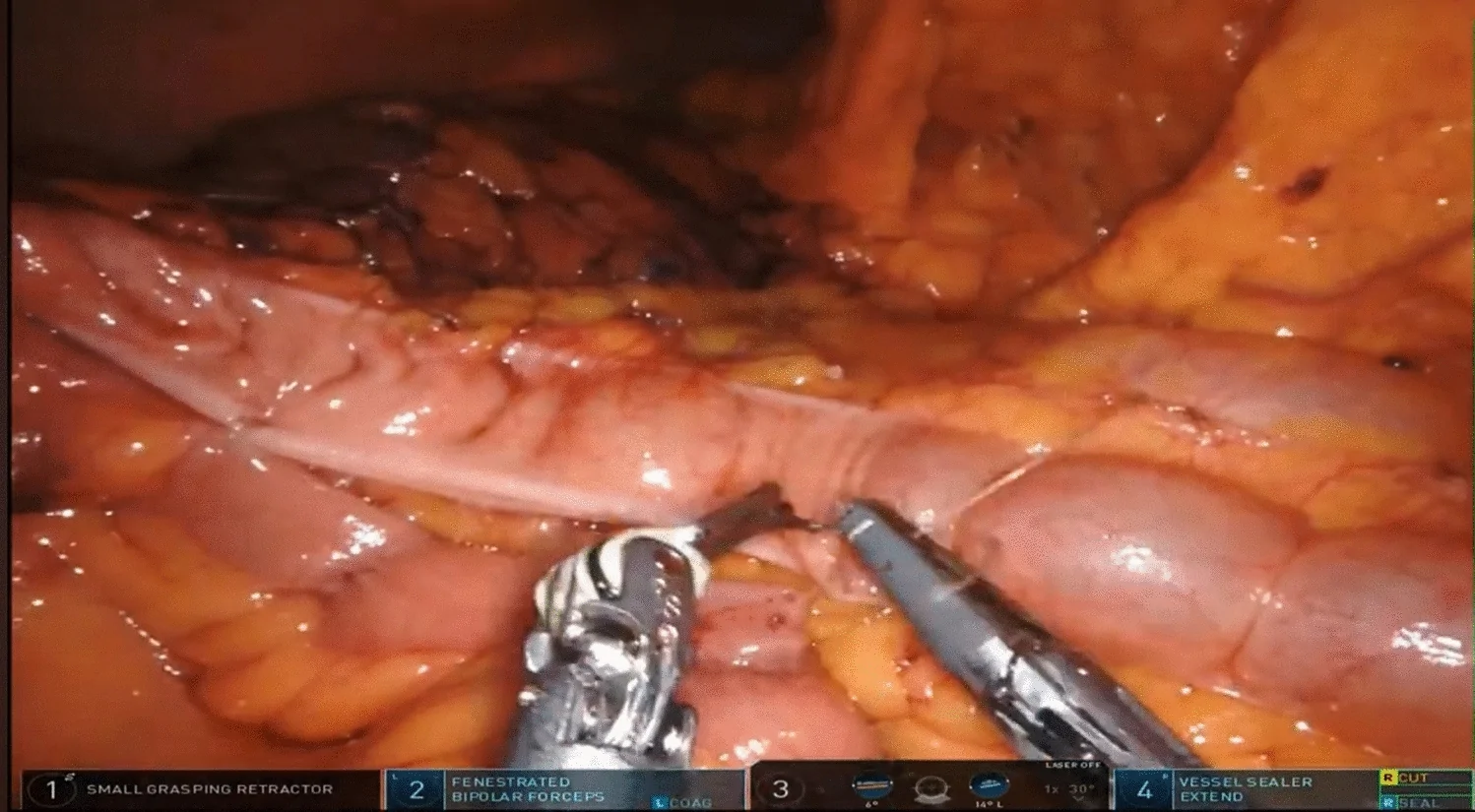
Robotic right colectomy: placing the anti-mesenteric stay sutures 6–8 cm apart

**Fig. 8:**
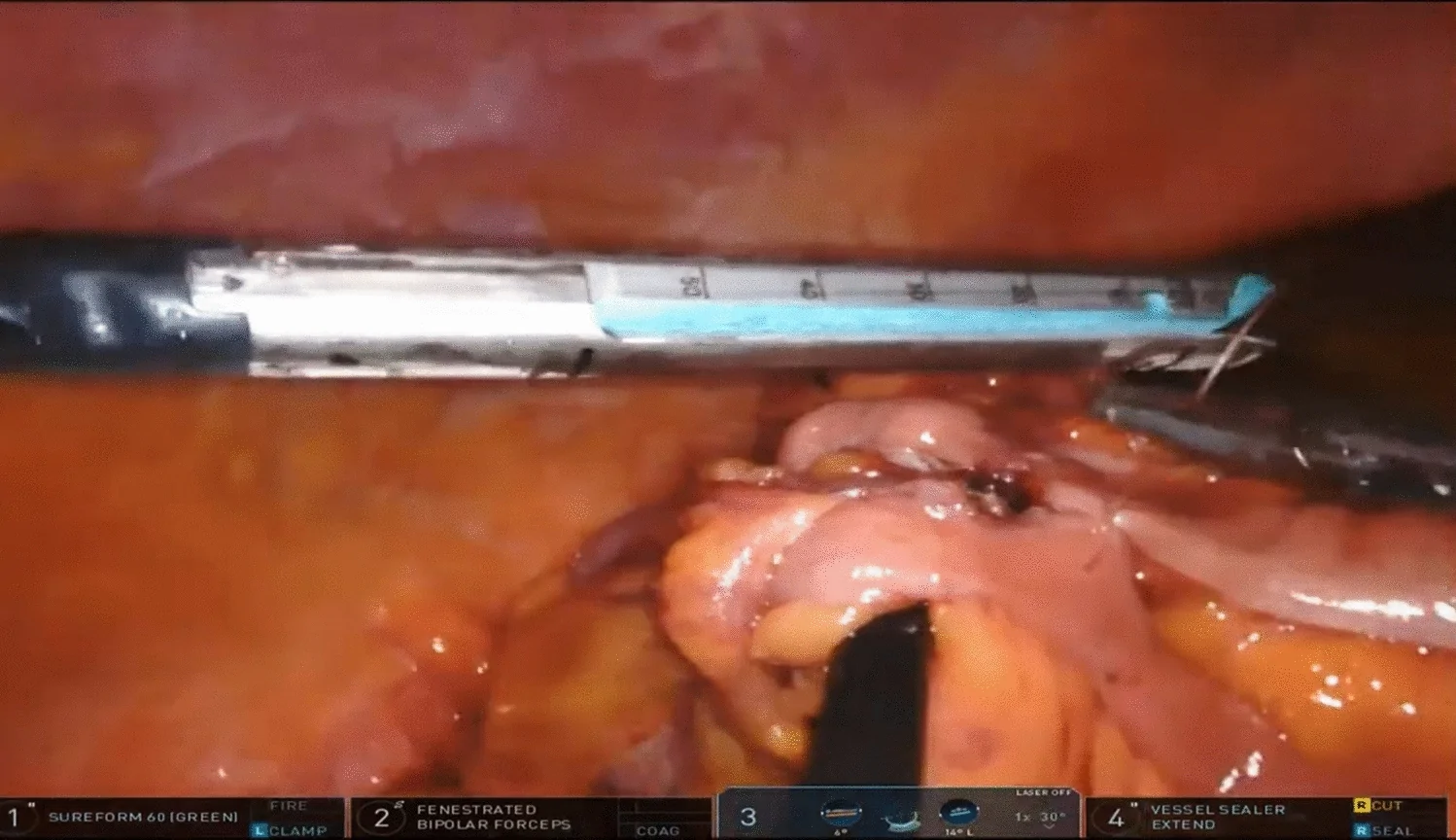
Robotic right colectomy: rotate the stapler so the large green jaw is lateral to facilitate easier insertion into the bowel

**Fig. 9:**
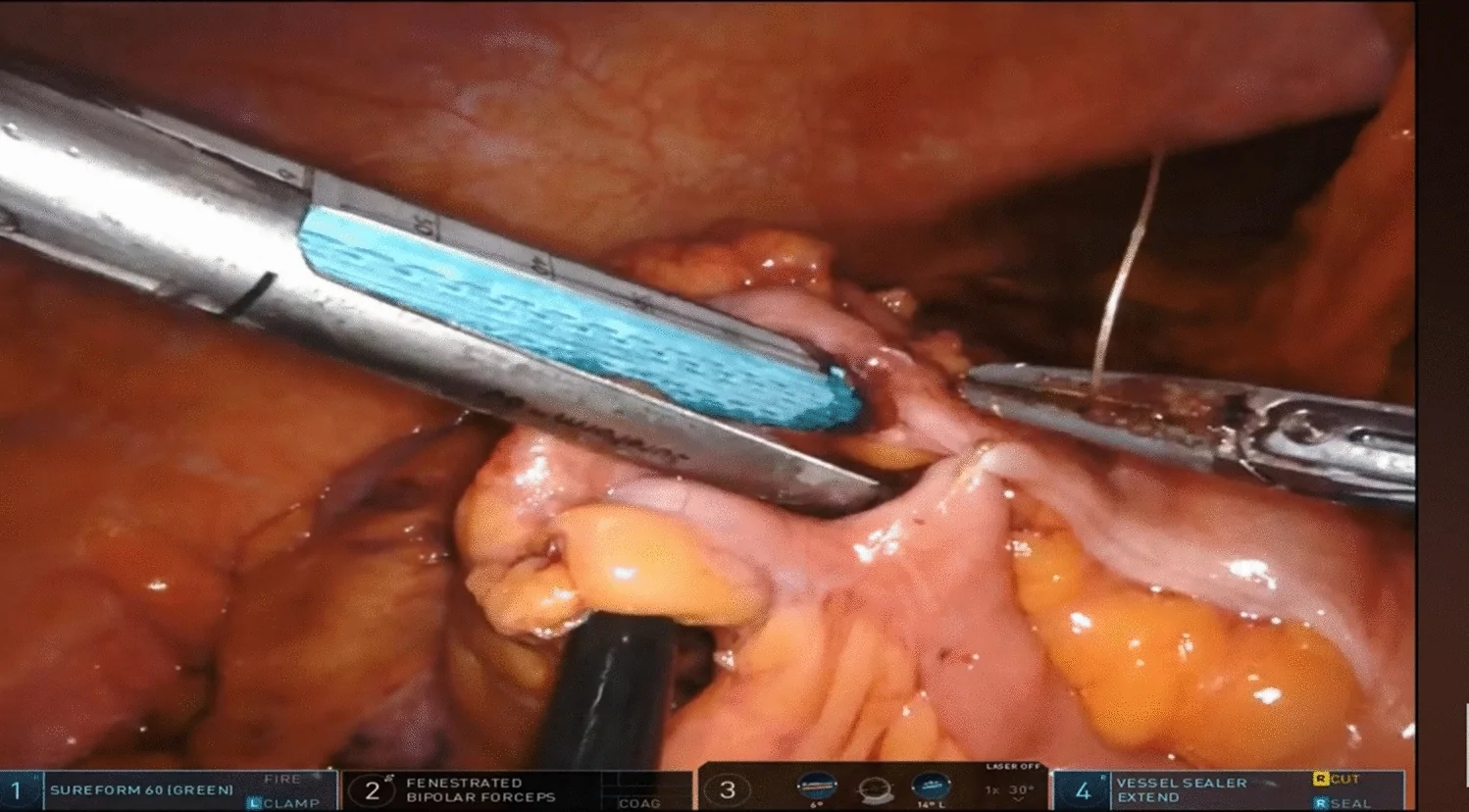
Robotic right colectomy: insert the metal jaw into the ileum first, then the large jaw into the colon

**Fig. 10:**
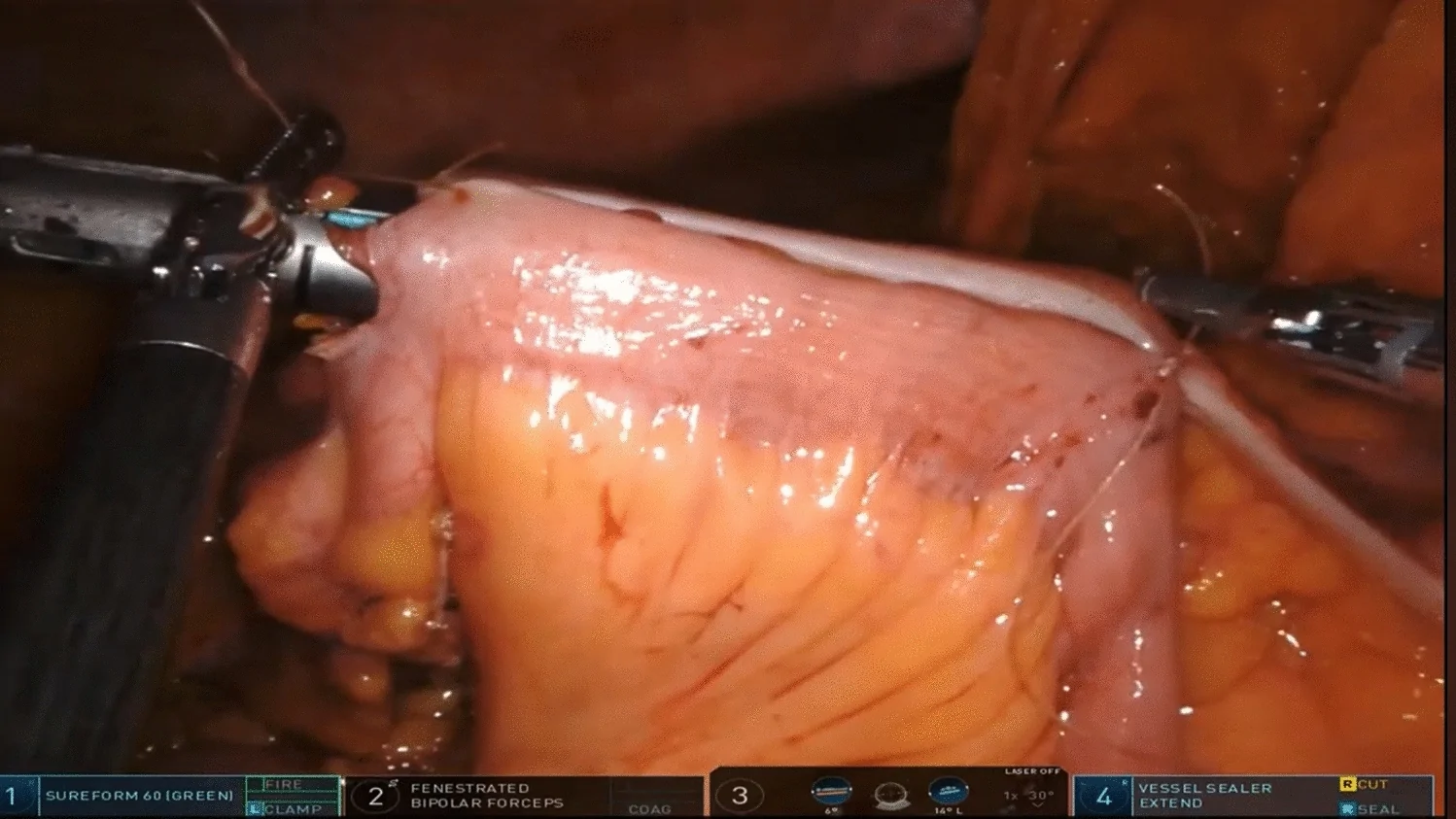
Robotic right colectomy: open stapler jaws elevated anteriorly so the mesentery will fall away prior to firing

**Fig. 11:**
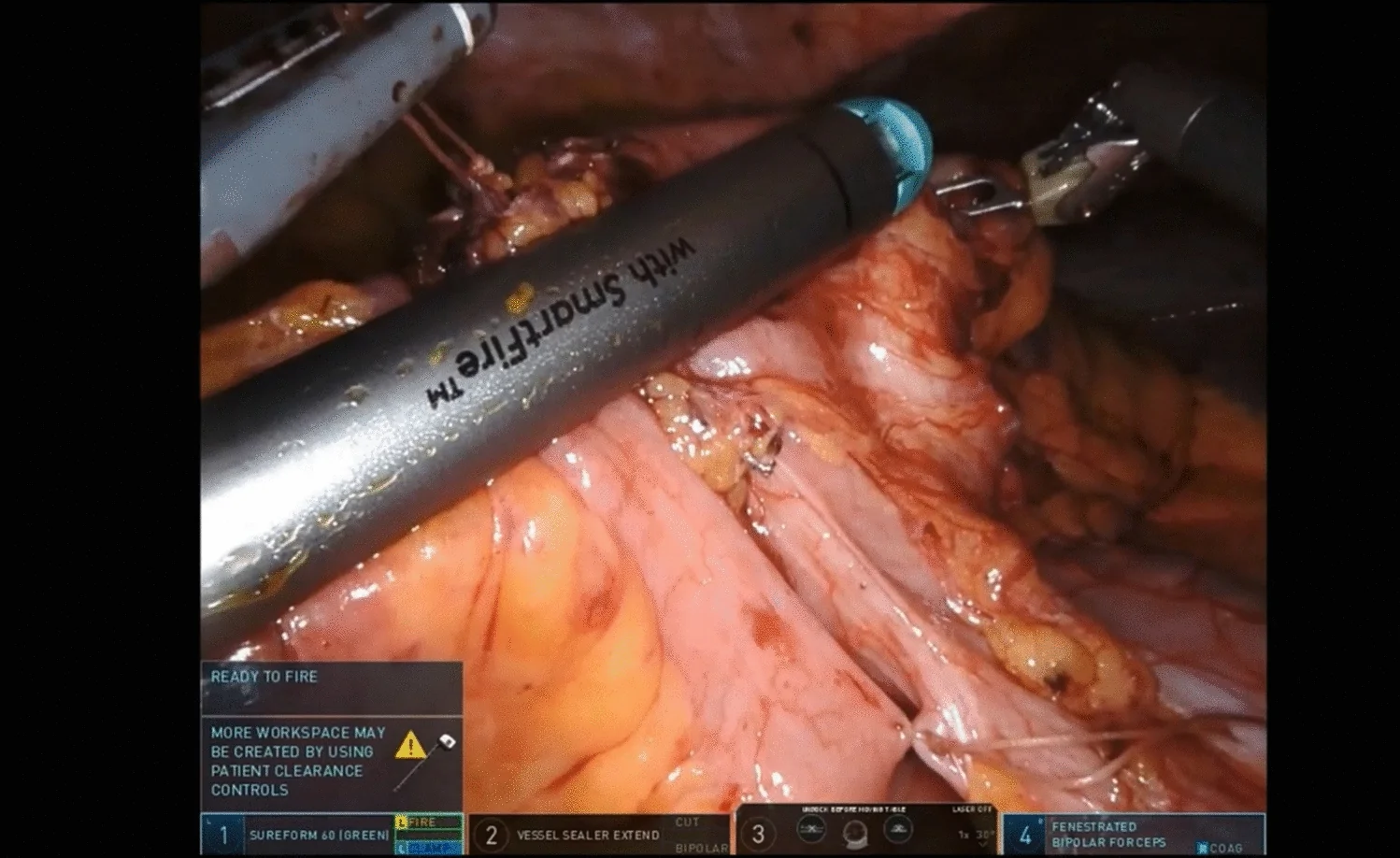
Robotic right colectomy: second stapler closing the enterocolostomy anastomosis. Note the stapler is placed just distal to the proximal stay suture and perpendicular to the first staple line

**Fig. 12:**
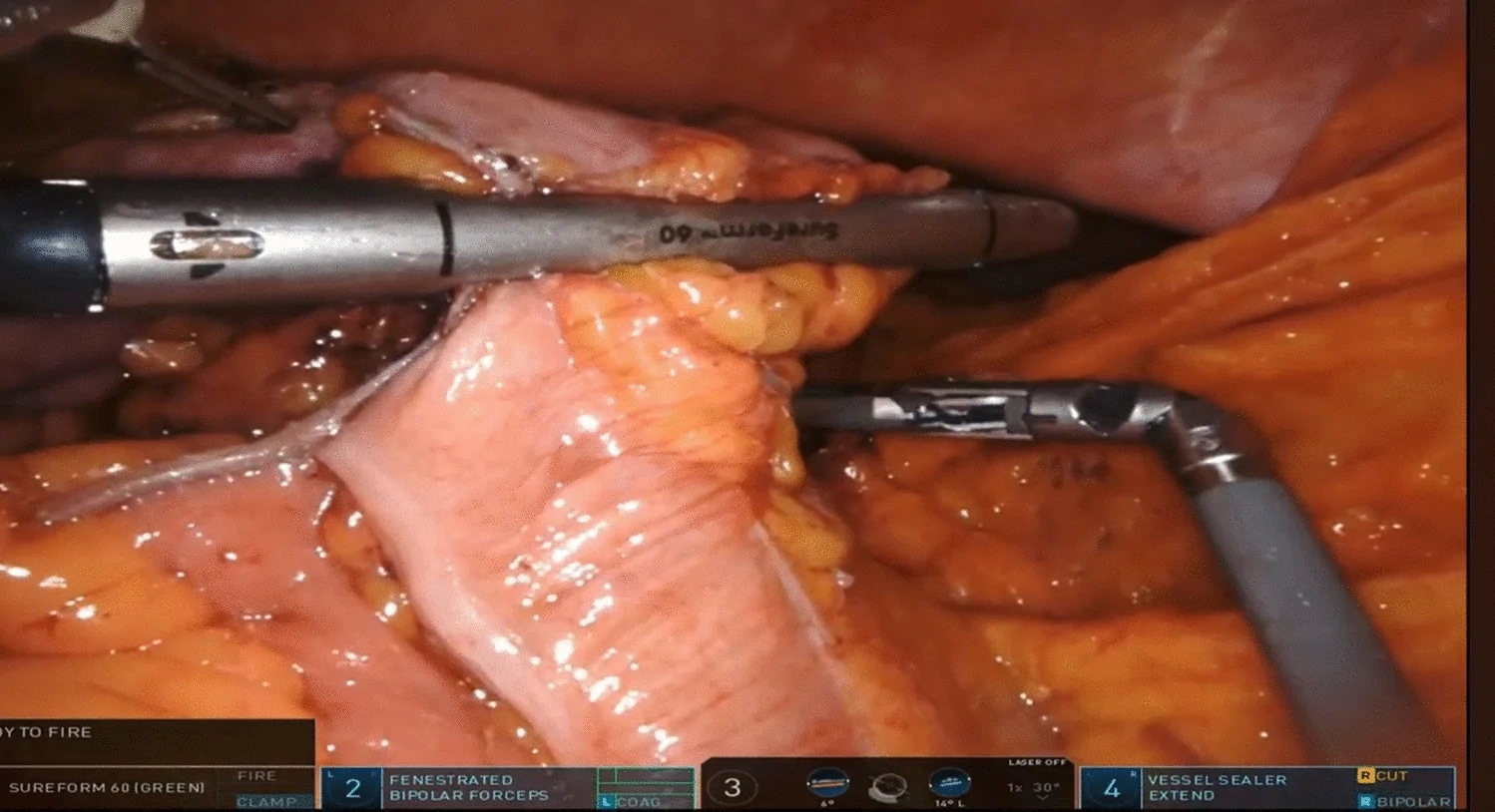
Robotic right colectomy: third stapler completing the closure of the ileocolostomy anastomosis. Note that the stapler is placed perpendicular to the second staple line to ensure correct staple-line closure without defects or ischemia

**Fig. 13:**
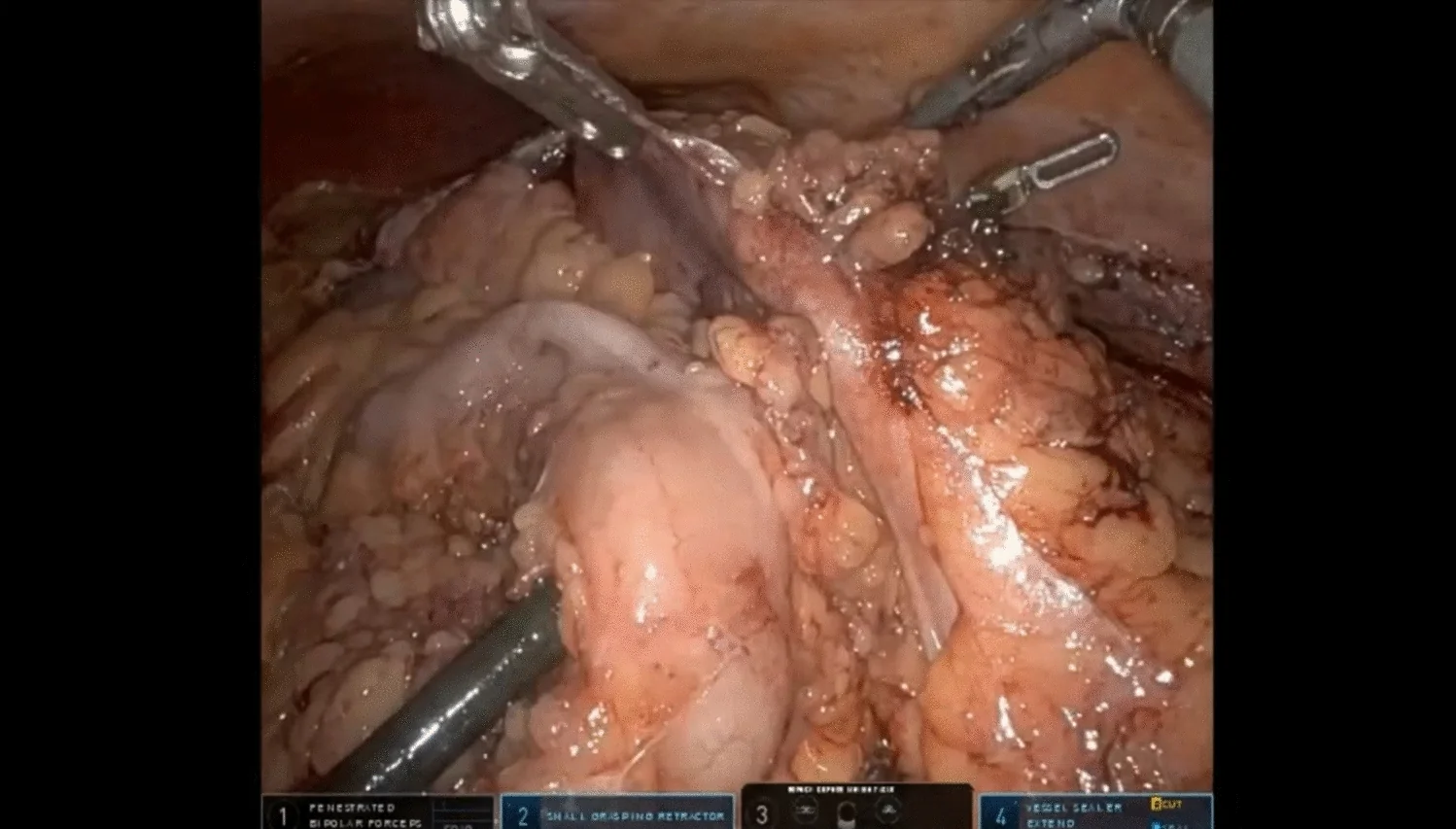
Robotic left colectomy: mid-transverse colon and proximal sigmoid colon aligned. Lower fenestrated bipolar arm denotes mesenteric defect for future closure staple line

**Fig. 14:**
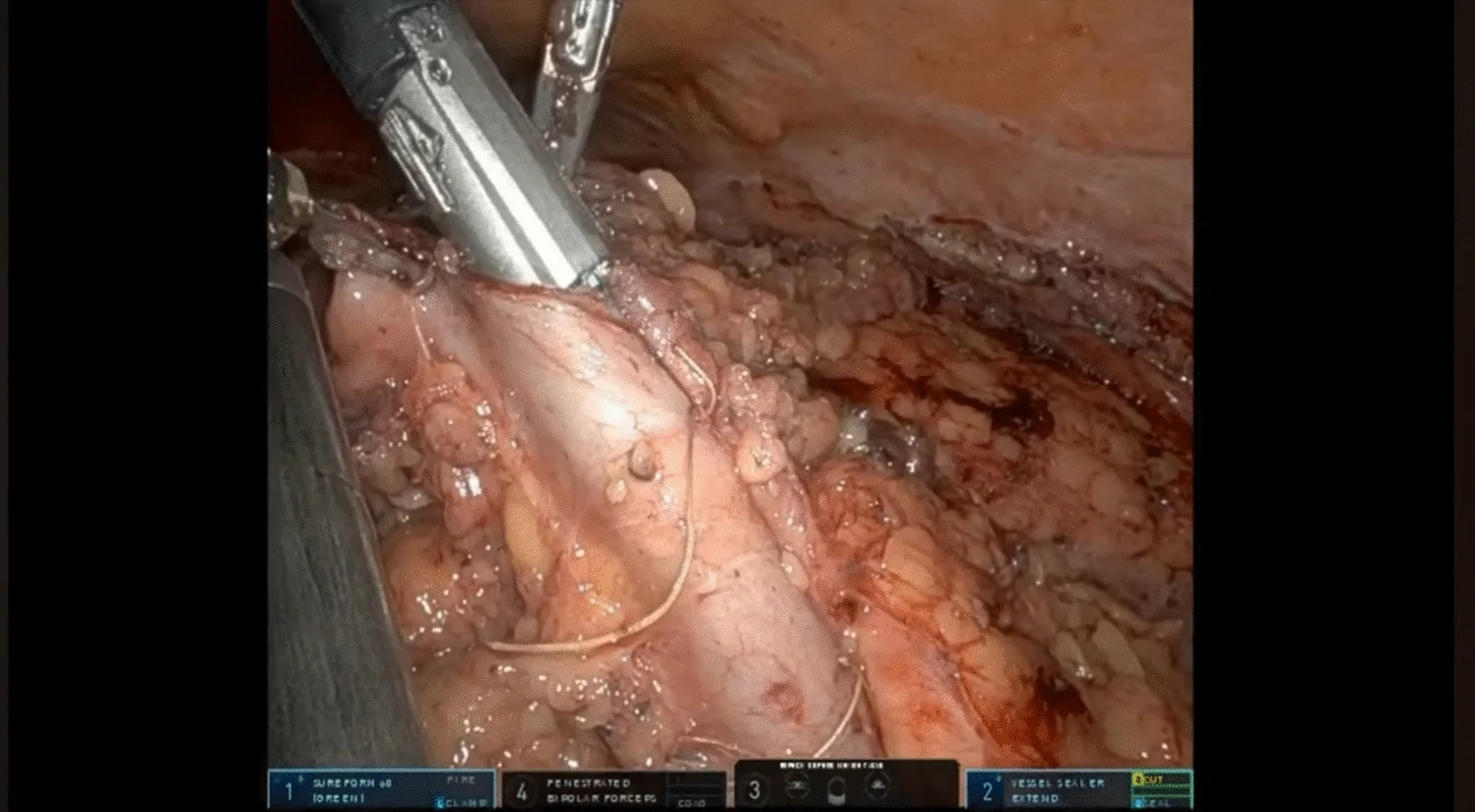
Robotic left colectomy: first stapler creating the colocolostomy anastomosis brought in via a LUQ 12-mm port

**Fig. 15:**
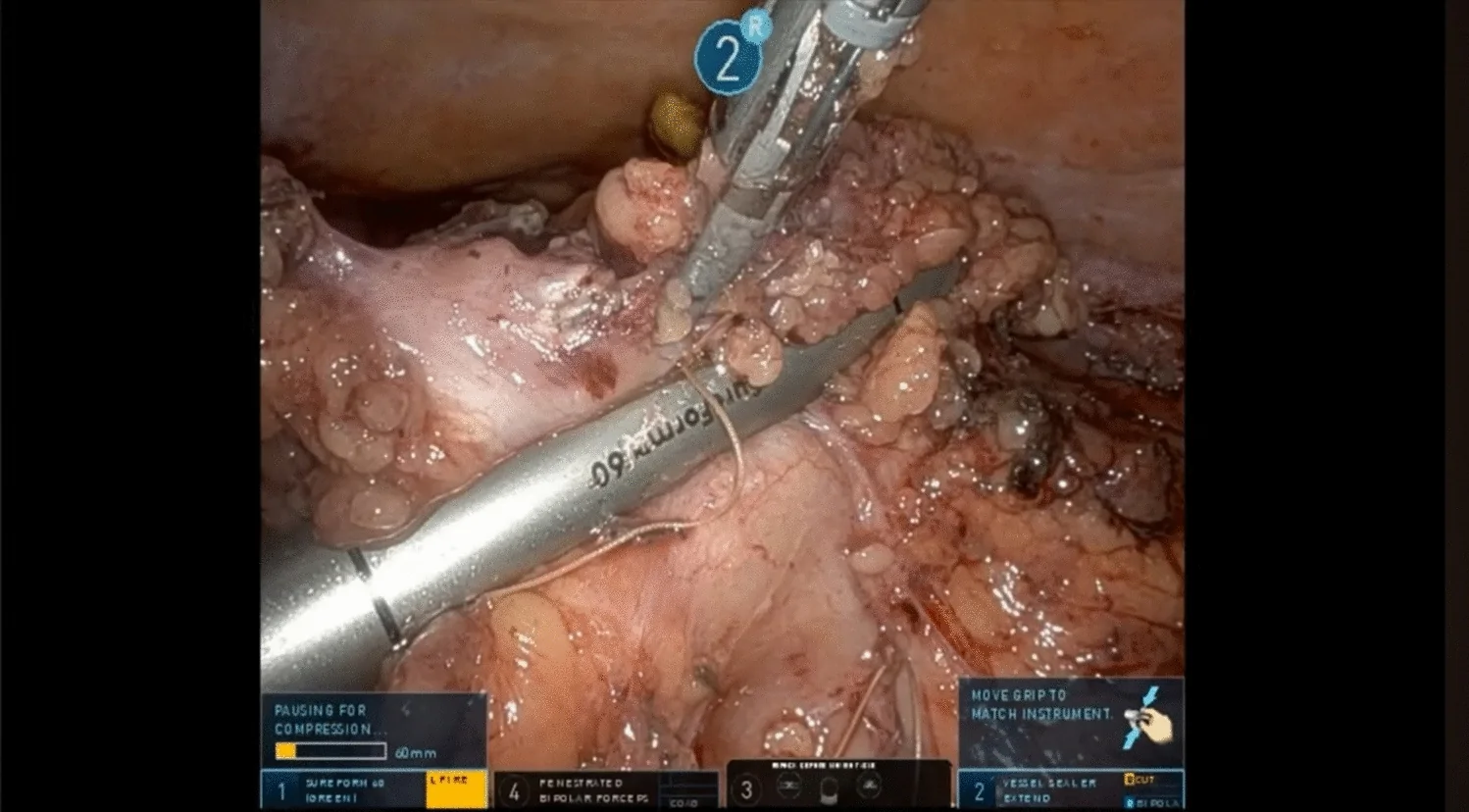
Robotic left colectomy: second stapler closing the colocolostomy anastomosis brought in via a RUQ 12-mm port. Note it is placed just distal to the first/proximal stay suture and perpendicular to the fist staple line

**Fig. 16:**
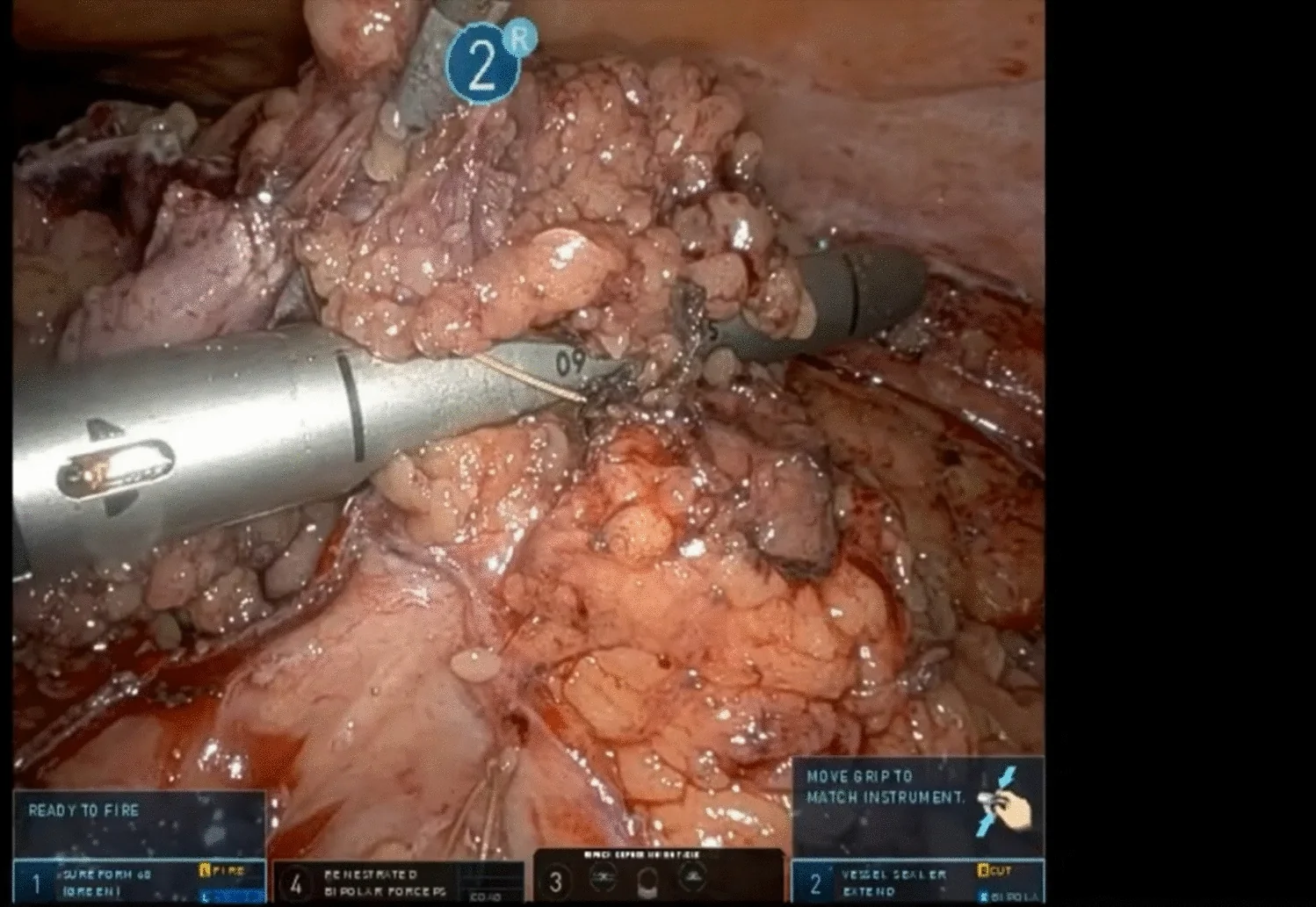
Robotic left colectomy: third stapler completing the closure of the colocolostomy anastomosis. Note that the stapler is placed perpendicular to the second staple line to ensure correct staple-line closure without defects or ischemia

**Fig. 17:**
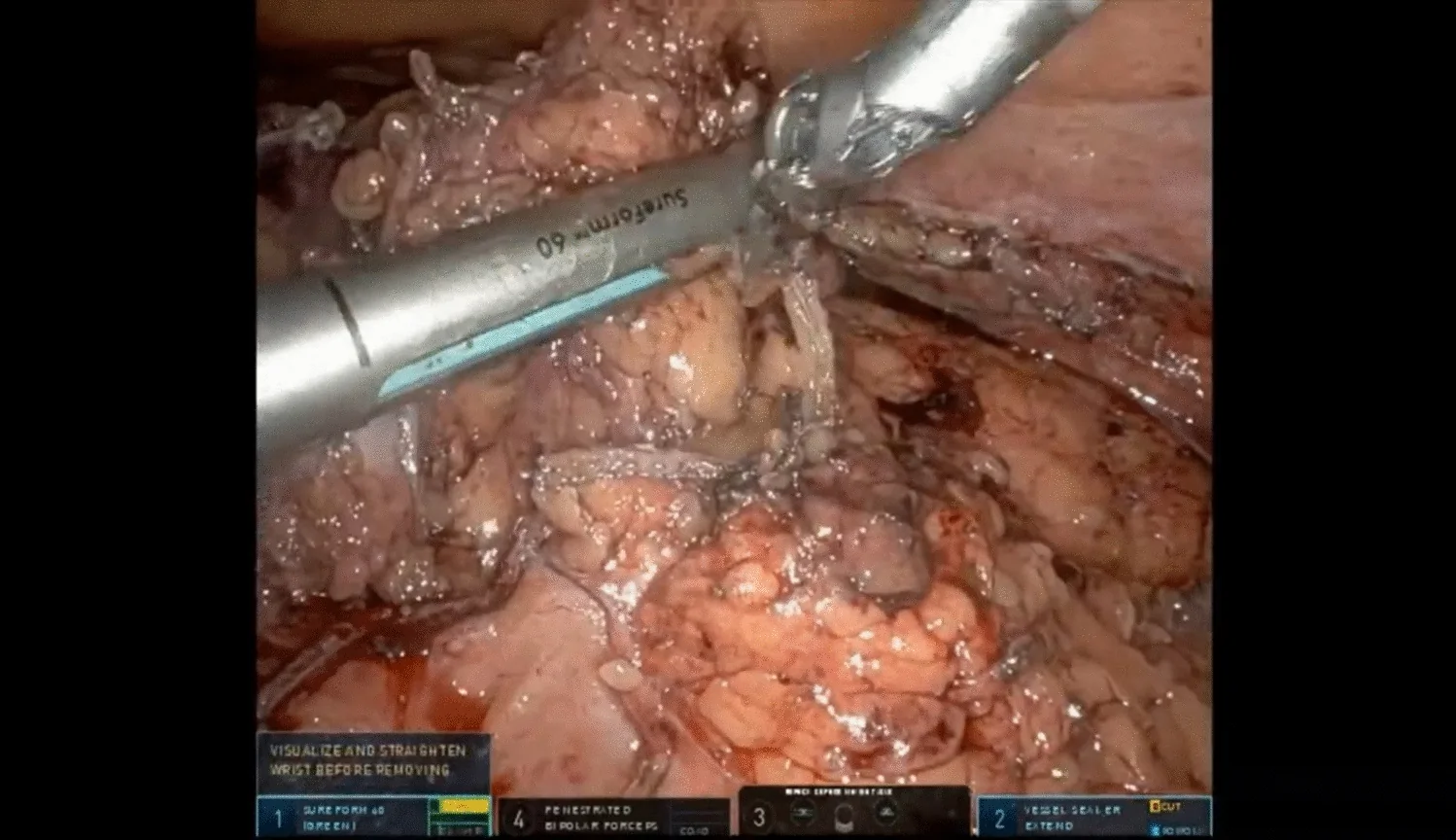
Robotic left colectomy: complete closure of the colocolostomy anastomosis. Note that the third staple-line completely closed the second staple line

**Fig. 18:**
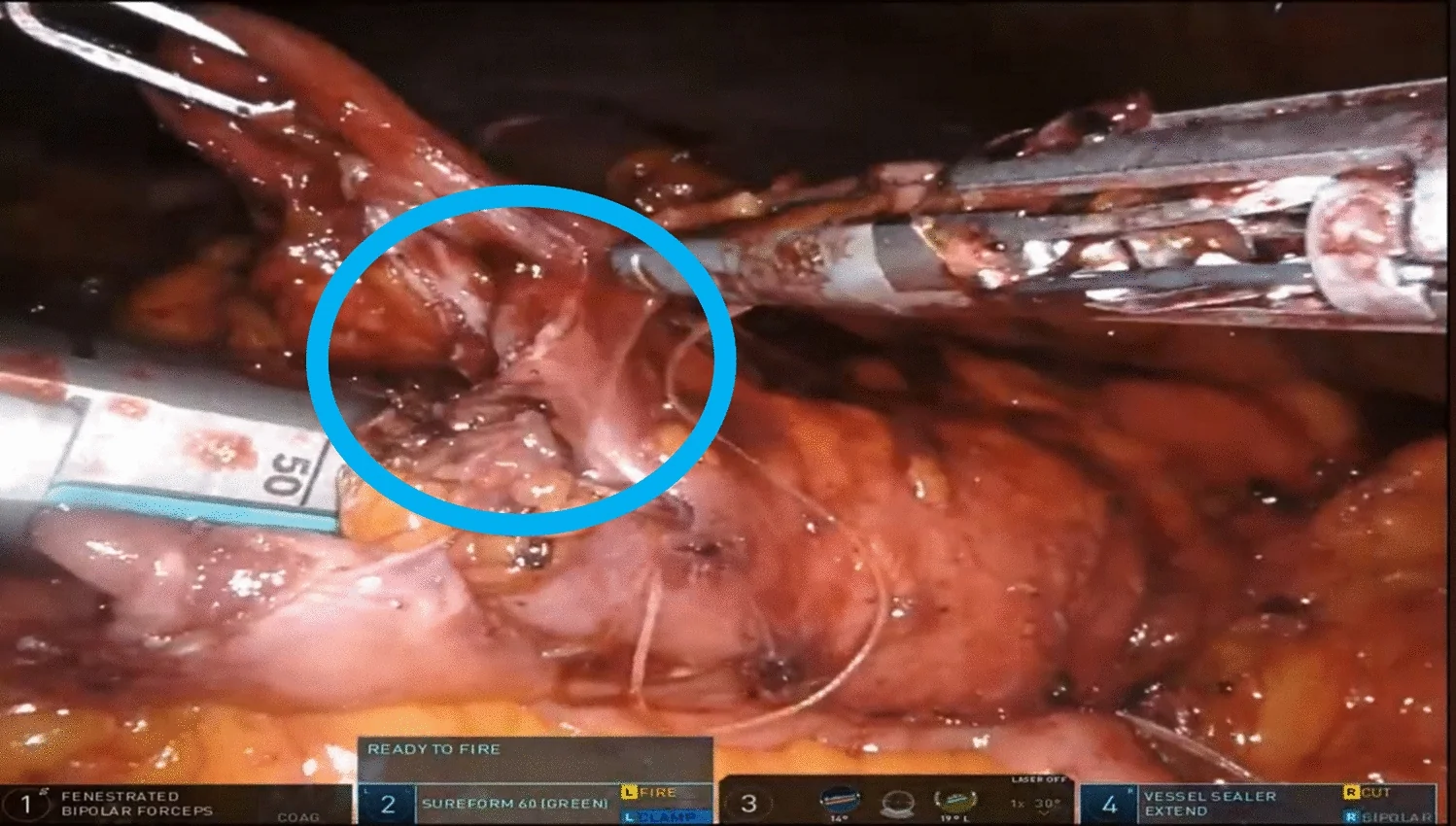
Robotic right colectomy: close inspection of the colon notes the large stapler jaw did not enter the colon lumen (blue circle) and needed to be removed and repositioned

**Fig. 19:**
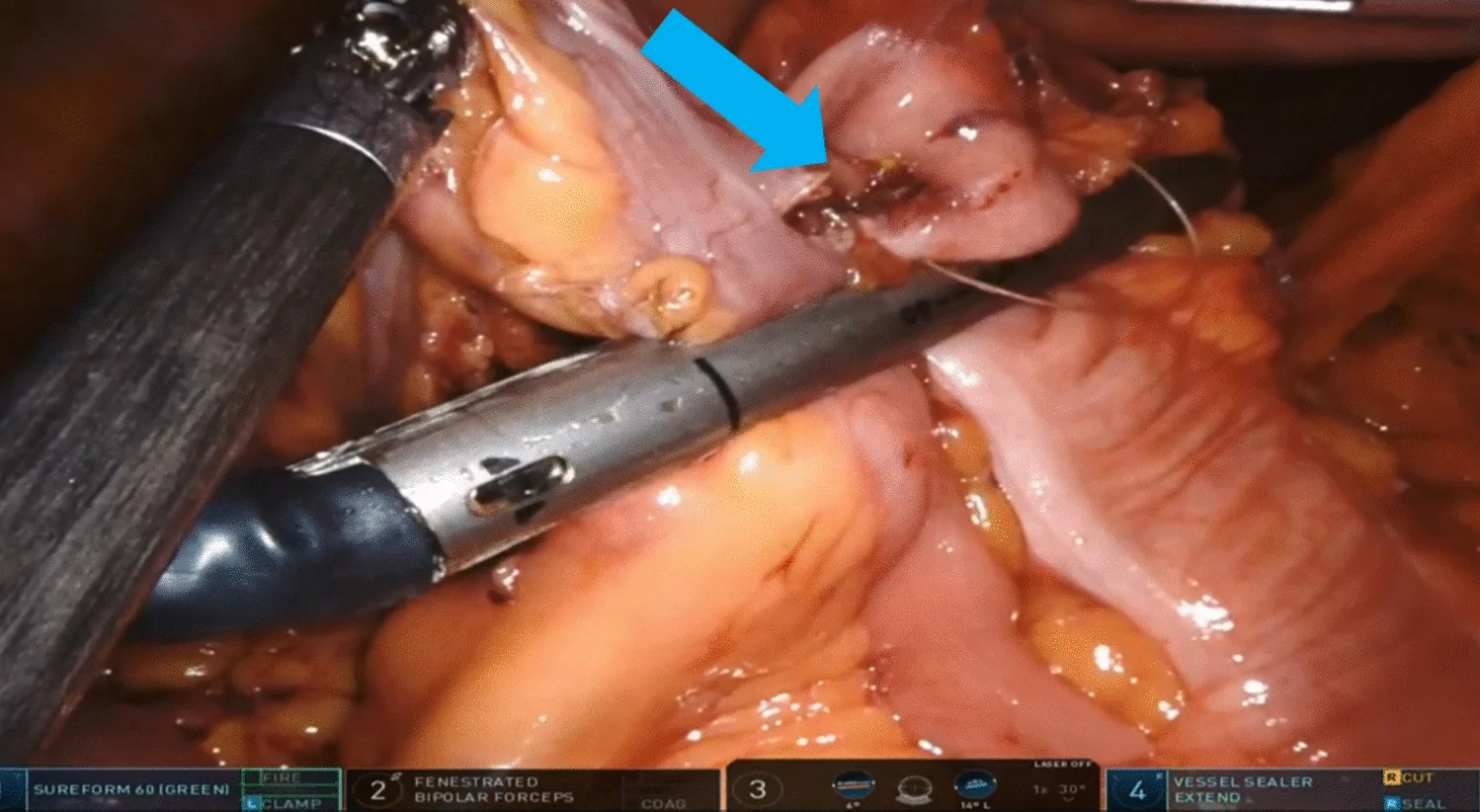
Robotic right colectomy: note the stapler is placed just distal to the proximal stay suture and perpendicular to the first staple line. Also note the common opening (blue arrow) is on the specimen side and not incorporated into the staple line

**Fig. 20:**
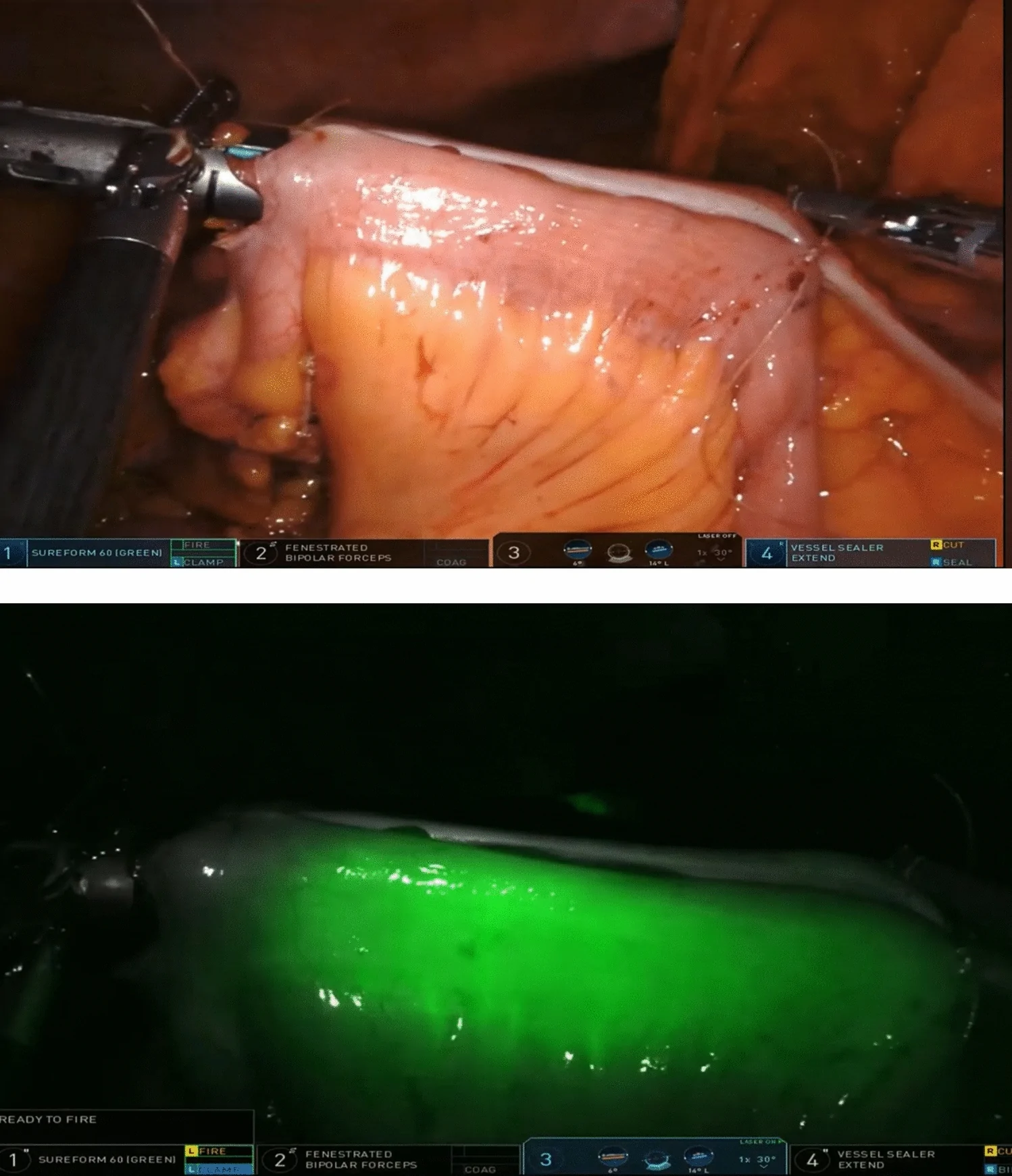
Robotic right colectomy: ICG evaluation of the anastomosis

**Fig. 21:**
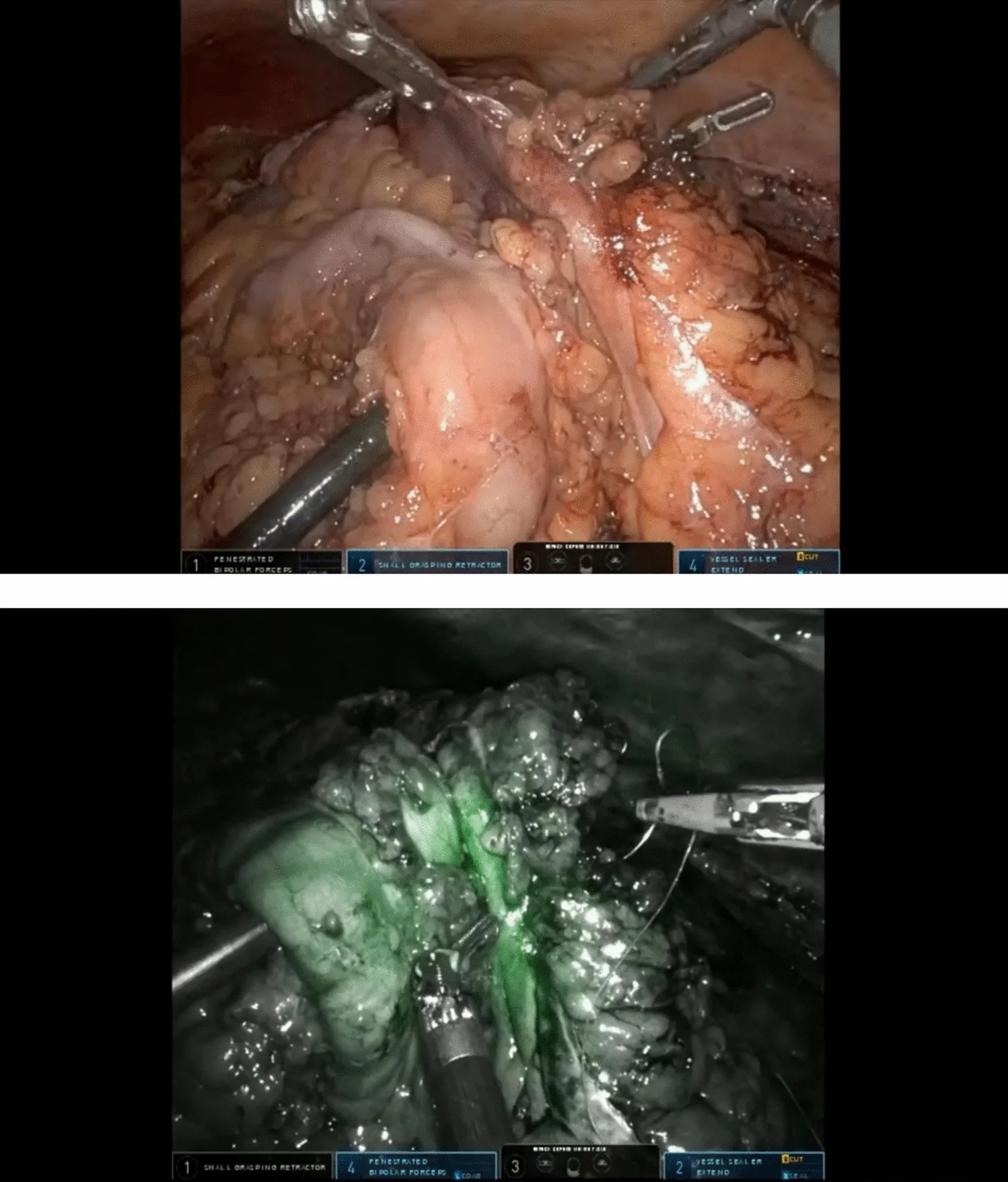
Robotic left colectomy: ICG evaluation of the planned anastomotic limbs

This arrangement ensures the necessary reach, orientation, and stapler angles required for consistent creation and closure of a double-stapled intracorporeal anastomosis. Once port placement and positioning are established, the procedure proceeds through a reproducible series of steps (Table 2), as follows:

1. Robot docking: Open the robotic arms widely at the boom for increased range of motion and decreased external collisions (Fig. 3). After docking, manually point each arm toward the abdomen and adjust to avoid collisions (Figs. 4 and 5).
2. Bowel mobilization and mesenteric division: Begin with complete mobilization of the bowel to include all attachments and vascular pedicles. The mesentery of the bowel is then fully divided to the planned specimen transection sites (Fig. 6). Complete skeletonization of the mesentery and epiploic fat is essential, particularly in left-sided resections where mesenteric bulk and colonic epiploica are more pronounced. This prevents inadvertent inclusion of mesenteric tissue within the staple line, which reduces the risk of bleeding and improves staple formation.
3. Stay suture placement: Place two anti-mesenteric stay sutures at the planned anastomotic site (Fig. 7). The proximal suture guides the removal of devascularized tissue during closure. The second suture, positioned 6–8 cm distal, provides traction, maintains orientation, and elevates both bowel limbs, allowing the mesentery to fall away during stapler introduction and serving as a crotch/anti-obstruction stitch. These sutures promote safe tissue handling and enhance reproducibility.
4. Enterotomy creation: Create an anti-mesenteric enterotomy on the afferent limb specimen side (ileum for right colectomy or mid-transverse colon for left colectomy). Create a corresponding enterotomy on the efferent limb specimen side, slightly medial to the anti-mesenteric border, between the mesenteric edge and the anti-mesenteric line. These enterotomies are positioned to optimize stapler trajectory through the available 12-mm port.
5. Anastomosis construction (double-stapled technique):
6. First stapler firing (anastomotic lumen): Insert a 60-mm enteric stapler (typically a green load) through the appropriate 12-mm port (RLQ for right colectomy, LUQ for left colectomy). Rotate the stapler 180° to orient the larger jaw laterally (Fig. 8). Guide the thin metal jaw into the afferent bowel and the thicker load jaw into the efferent bowel (Fig. 9). Lift the open stapler anteriorly toward the abdominal wall to ensure the mesentery falls away and is not incorporated into the anastomosis (Fig. 10). Close and fire the stapler to create the anastomotic lumen (Fig. 10). Inspect the anastomotic lumen for bleeding or ischemia.
7. Second stapler firing (common channel closure): Insert a second 60-mm enteric stapler and rotate it 180° again to orient the larger jaw laterally. Guide the stapler with the thicker jaw positioned just distal to the first/proximal stay suture and perpendicular to the first staple line (Figs. 11, 12, 13, 14, and 15). Close and fire the stapler (Figs. 12, 16, 17, and 18). Ensure the common opening is on the specimen side and not incorporated into the staple line (Fig. 19).
8. Perpendicularity and additional firings: Maintaining perpendicularity of the staple lines is essential to avoid gaps, ischemia, or narrowing. If an additional staple load is required to complete the common channel closure due to the width of the combined bowel, ensure it is fired perpendicular to the previous staple line to avoid creating a hole or ischemia at any crossing points (Fig. 12 and 17).
9. Perfusion assessment: Indocyanine green (ICG) fluorescence imaging can be used to assess tissue perfusion at the anastomotic site before and after stapler firing (Figs. 20 and 21).
10. Specimen retrieval: The resected specimen can then be placed into an endocatch bag and retrieved from your planned extraction location.

**Table 2:** Minimally invasive fully stapled intracorporeal side-to-side anastomosis steps

Side-to-side intracorporeal double-stapled anastomosis
1) Open the arms widely at the boom (Fig. 3).
2) Point the docked ports toward the side of the pathology (Figs. 4 and 5).
3) Mobilize the bowel completely to include all attachments and all vascular pedicles
4) Divide the mesentery of the bowel completely to the planned specimen transection sites (Fig. 6).
5) Orient the bowel next to each other and place an anti-mesenteric stay suture at the visible line of vascular demarcation
6) Place a second anti-mesenteric stay suture 6–8 cm distal to the first one (Fig. 7).
7) Create an anti-mesenteric enterotomy on the afferent limb specimen side
8) Create an enterotomy on the efferent limb specimen side between the mesenteric edge and anti-mesenteric line
9) Insert a 60-mm stapler into the right-lower-quadrant port and rotate it 180° so that the staple-load jaw is lateral (Fig. 8).
10) Guide the thin metal jaw of the stapler into the afferent bowel and thicker load jaw into the efferent bowel (Fig. 9).
11) Lift the stapler anteriorly so that the mesentery falls away and is not incorporated into the anastomosis (Fig. 10).
12) Close and fire the stapler (Figs. 10 and 14).
13) Inspect the anastomotic lumen to ensure there is no bleeding or ischemia
14) Insert a second stapler and again rotate it 180° to orient the larger jaw laterally
15) Guide the stapler with the thicker jaw going posteriorly so that is just distal to your first/proximal stay suture and perpendicular to the first staple line (Figs. 11 and 15).
16) Close and fire the stapler
17) If an additional staple load is required to complete the closure, ensure it is perpendicular to the second staple line to avoid creating a hole or ischemia at the crossing staple lines (Fig. 12 and 16).
18) The specimen can then be placed into an endocatch bag and retrieved from your planned extraction location
19) Perform a leak test (e.g., air leak test with submersion) for left-sided anastomoses

## Practical considerations/discussion

Successful adoption of this fully stapled ICA technique requires attention to several practical considerations and potential pitfalls:

- Candidate selection: This technique is generally well suited for most patients undergoing minimally invasive right or left colectomy. However, it may be less ideal for cases with poor tissue quality warranting manual inspection of bowel integrity or for bulky masses requiring multivisceral resection, where the constraints of stapler placement or visualization might be compromised.
- Patient positioning: A common pitfall in MIS colectomy is inadequate exposure. It is important to utilize steep trendelenburg and side tilt just to the point of adequate exposure to perform the operation. There are no set degrees for table position. Positioning is patient specific based on volume and position of viscera relative to the colon. Intraabdominal adiposity often requires steeper positioning. The key for right colectomy is exposure of the ileocolic and middle colic pedicles and slight trendelenburg is often enough to achieve adequate exposure. For left colectomy, exposure of the IMV is key.
- Mesenteric skeletonization: Complete skeletonization of the bowel and removal of epiploic fat are paramount. Inadvertent inclusion of mesenteric tissue or fat within the staple line can lead to bleeding, compromised staple formation, and an increased risk of anastomotic complications (Videos 1 and 2). Lifting the open stapler anteriorly to allow the mesentery to fall away is a crucial step to prevent this (Video 2).
- Stapler orientation and luminal placement: Proper orientation of the stapler is critical. The thin jaw should enter the afferent bowel, and the thicker jaw should enter the efferent bowel, allowing for natural tissue roll-in without torque or twisting. Confirming full intraluminal placement of both jaws before firing is essential; if there is any doubt regarding lumen entry or alignment, the stapler should be withdrawn and repositioned (Fig. 18) (Videos 2 and 3).
- Perpendicular firing and avoiding crossing staple lines: When closing the common enterotomy, the second staple line must be fired perpendicular to the first anastomotic staple line. This ensures a clean, non-obstructing common channel and avoids creating areas of ischemia or narrowing. If multiple firings are needed for closure, maintaining perpendicularity is key (Figs. 12, 16, 17) (Videos 1 and 3). Improper alignment can lead to a “rolling away” of the colon, resulting in a non-perpendicular staple line that narrows the lumen (Video 3). Careful adjustment and elevation of the bowel are necessary to achieve proper orientation before firing.
- Stay sutures: Anti-mesenteric stay sutures, placed 6–8 cm apart, are invaluable for traction, maintaining optimal orientation of the bowel limbs, and facilitating stapler introduction and alignment. They help keep the mesentery out of the stapler path and can be used to manipulate the bowel for better visualization and approximation (Videos 1 and 2).
- Leak test: While not explicitly part of the stapling sequence, it is *imperative* to routinely perform a leak test (e.g., air leak test with submersion in saline or flexible endoscopy with air insufflation) for all colorectal anastomoses, especially left-sided ones, at the conclusion of the anastomosis to confirm integrity before abdominal closure.
- Cost implications: While the technique aims for efficiency and reproducibility, the reliance on multiple stapler loads can have cost implications compared to hand-sewn anastomoses. This is a factor for healthcare systems to consider in adoption.
- Laparoscopic vs. robotic approach: While the fundamental principles are transferable, the robotic platform offers enhanced 3D visualization, tremor filtration, and superior instrument articulation that facilitate precise stapler placement and manipulation in challenging anatomical situations. These advantages can contribute to a smoother learning curve and higher reproducibility for the robotic execution compared to a purely laparoscopic approach.

## Conclusion

As intracorporeal anastomosis becomes more prevalent in minimally invasive colorectal surgery, reproducible techniques with efficient workflows are critical to improve outcomes and encourage adoption. The double-stapled intracorporeal side-to-side anastomosis described here provides a standardized, stapler-based approach that parallels traditional extracorporeal techniques, while eliminating intracorporeal hand-sewn closure. This technique is applicable to both right and left colectomies and offers the potential for reproducibility across laparoscopic and robotic platforms. By leveraging a technique already well understood by minimally invasive surgeons, this approach aims to facilitate technical trust and lower the barrier to adoption of intracorporeal anastomosis, thereby promoting its wider implementation and teaching. Future studies, including prospective comparative trials, are warranted to investigate the learning curve, definitive safety profile, and long-term clinical outcomes of this technique in human patients.

## Supplementary Information

Below is the link to the electronic supplementary material.

Supplementary file1 (DOCX 9 KB)
