# Supplementary material for: Double-stapled intracorporeal anastomosis for minimally invasive right and left colectomies: a standardized technical approach
Source: Surg Endosc. 2026 Oct 1;40(11):10108–20. doi: 10.1007/s00464-026-13360-w (PMC13754767; doi:10.1007/s00464-026-13360-w)
Supplement: Supplementary file 1 [file 464_2026_13360_MOESM1_ESM.docx]

**Supplemental Material - Videos**

**Practical Considerations:**

**Video 1:** Fine Tuning Robotic Double-Stapled Intracorporeal Ileocolonic Anastomosis

<http://www.youtube.com/watch?v=7SpUTVxYTeM>

**Video 2:** Taking the mesentery - Double-stapled robotic intracorporeal ileocolonic anastomosis

<http://www.youtube.com/watch?v=LUlck4lZDqQ>

**Video 3:** Beware The Colon Will Roll - Robotic Double-Stapled Intracorporeal Ileocolonic Anastomosis.

<http://www.youtube.com/watch?v=RYmDSVpvnxM>

**Full technique videos:**

**Video 4:** A Fully Double Stapled Right Colectomy Intracorporeal Anastomosis

<https://www.youtube.com/watch?v=C9B5VEl9YME>

**Video 5:** Left colo-colonic fully stapled robotic intracorporeal anastomosis

<http://www.youtube.com/watch?v=BA6u9ZxLz4I>
